# A 25-year Experience at an Academic Medical Center in the United States: Are There Racial Disparities in the Prognosis of Patients Diagnosed With Hypopharyngeal Carcinoma?

**DOI:** 10.7759/cureus.11306

**Published:** 2020-11-03

**Authors:** Toms Vengaloor Thomas, Kati Krishna, Hiba Z Ahmed, Eswar Mundra, Anu Abraham, Eldrin Bhanat, Mary R Nittala, Satya Packianathan, Srinivasan Vijayakumar

**Affiliations:** 1 Radiation Oncology, University of Mississippi Medical Center, Jackson, USA; 2 Pathology, University of Mississippi Medical Center, Jackson, USA; 3 Orthopaedic Surgery, University of Mississippi Medical Center, Jackson, USA

**Keywords:** hypopharyngeal carcinoma, racial disparities

## Abstract

Introduction

This study attempted to identify disparities in outcomes between African American (AA) and Caucasian American (CA) patients treated for hypopharyngeal carcinoma at a tertiary care institution over the past 25 years.

Methods

An institutional review board (IRB)-approved and Health Insurance Portability and Accountability Act (HIPPA)-compliant retrospective analysis was performed on patients with squamous cell carcinoma of the hypopharynx treated at our institution between January 1994 and December 2018. Data regarding demographics, stage, treatment, and follow-up were collected. Outcomes, including median survival and overall survival, were calculated using the Kaplan-Meier method. All analyses were performed using the Social Packages for the Social Sciences (SPSS) v. 24 (IBM Corp., Armonk, NY).

Results

We identified 144 hypopharyngeal carcinoma patients who were treated during this period. Our patient cohort consisted of 61.8% AA and 35.4% CA (P=0.538). Overall, 96% of them presented at an advanced stage (Stages III & IV) of the disease, and only 4% presented in the early stages (Stages I & II). There was no significant difference between AA and CA patients who presented with advanced disease (96.6% vs. 94.1%).

In our patient cohort, 15.3% of patients did not receive any therapy; however, 51.4%, 22.9%, and 10.4% of them underwent definitive chemoradiotherapy, definitive surgery, or palliative chemotherapy, respectively. There were no significant differences in patient racial proportions within each treatment group.

The median follow-up of the entire cohort was 13 months. There was no significant difference between the median survival of AA and that of CA patients (16 months vs. 15 months; p=0.917). Moreover, there was no significant difference in the overall survival between AA and CA patients at three years (27.2% vs. 36.3%; p=0.917) and five years (20.4 % vs. 16.7 %; p=0.917).

Conclusions

A retrospective review of patients with hypopharyngeal cancer treated at our institution over the previous 25 years did not identify significant racial disparities regarding the stage at presentation or prognosis. This study suggests that when patients have equal access to care, they appear to have a similar prognosis despite racial differences. Further studies are needed to validate this hypothesis.

## Introduction

Hypopharyngeal cancer is a rare malignancy presenting mostly in the advanced stages partly due to its anatomic location and delayed symptomatic presentation. Extensive research into racial disparities in head and neck cancers exists, but racial disparities in hypopharyngeal cancer have not been concretely identified due to the rarity of this disease [1-2].

In a New York University Oral Cancer Research Center study conducted between 1975 and 2002, the incidence of hypopharyngeal cancer was 1.4 and 0.8 per 100,000 for African American (AA) and Caucasian American (CA), respectively. In contrast, the mortality appeared to be higher among AA (0.2 vs. 0.1/100,000) [3]. Males tended to have higher incidence and mortality rates than females, and AA males had the highest overall reported mortality rates over 28 years [3]. Studies by Ragin et al. and Petersen et al. suggested that racial differences in the stage at diagnosis and treatment prognoses between AA and CA patients may underlie these disparities in survival outcomes [1,3-4].

As the only academic medical center and safety-net hospital in Mississippi, the University of Mississippi Medical Center (UMMC) has treated a significant number of hypopharyngeal cancer patients [5]. The purpose of our study was to determine if racial disparities existed in outcomes between AA and CA patients treated for hypopharyngeal carcinoma at this tertiary care institution over the past 25 years.

## Materials and methods

The University of Mississippi Medical Center (UMMC) Institutional Review Board (IRB) approved all the investigations. The written consent requirement was waived due to the retrospective nature of the study. Data were collected by reviewing the charts of patients diagnosed between January 1994 and December 2018 from the UMMC Head and Neck Cancer Database. Research electronic data capture (RedCap) was utilized to gather and store the patient's information in password-protected computers.

We identified 144 patients with hypopharyngeal cancer treated at our institution during our study period. We collected patient demographic data, including age, sex, race, smoking history, alcohol use history, insurance status, aspiration risk and weight at diagnosis, and tumor characteristics data, including pathology, subsite, clinical-stage, and the pathological stage. Patients self-declared their race during the initial patient encounter and we collected this racial information from the patients' chart. The TNM classification system of the American Joint Committee on Cancer 7th Edition (AJCC 7) was used for staging. The data on treatment, including surgery, adjuvant radiation, adjuvant chemoradiation, definitive chemoradiation, salvage treatments, palliative chemotherapy, or hospice care, were also collected from the patient charts. The Mississippi Cancer Registry was used to collect the data on follow-up and vital statistics of the patients.

We used the Social Packages for the Social Sciences (SPSS) v. 24 software (IBM Corp., Armonk, NY) for data analysis. The Kaplan-Meier method was used to evaluate overall survival (OS), and the log-rank test compared the outcomes between the different treatment groups. The patients who were alive at the time of the last follow-up were categorized as censored cases.

## Results

**Figure 1:**
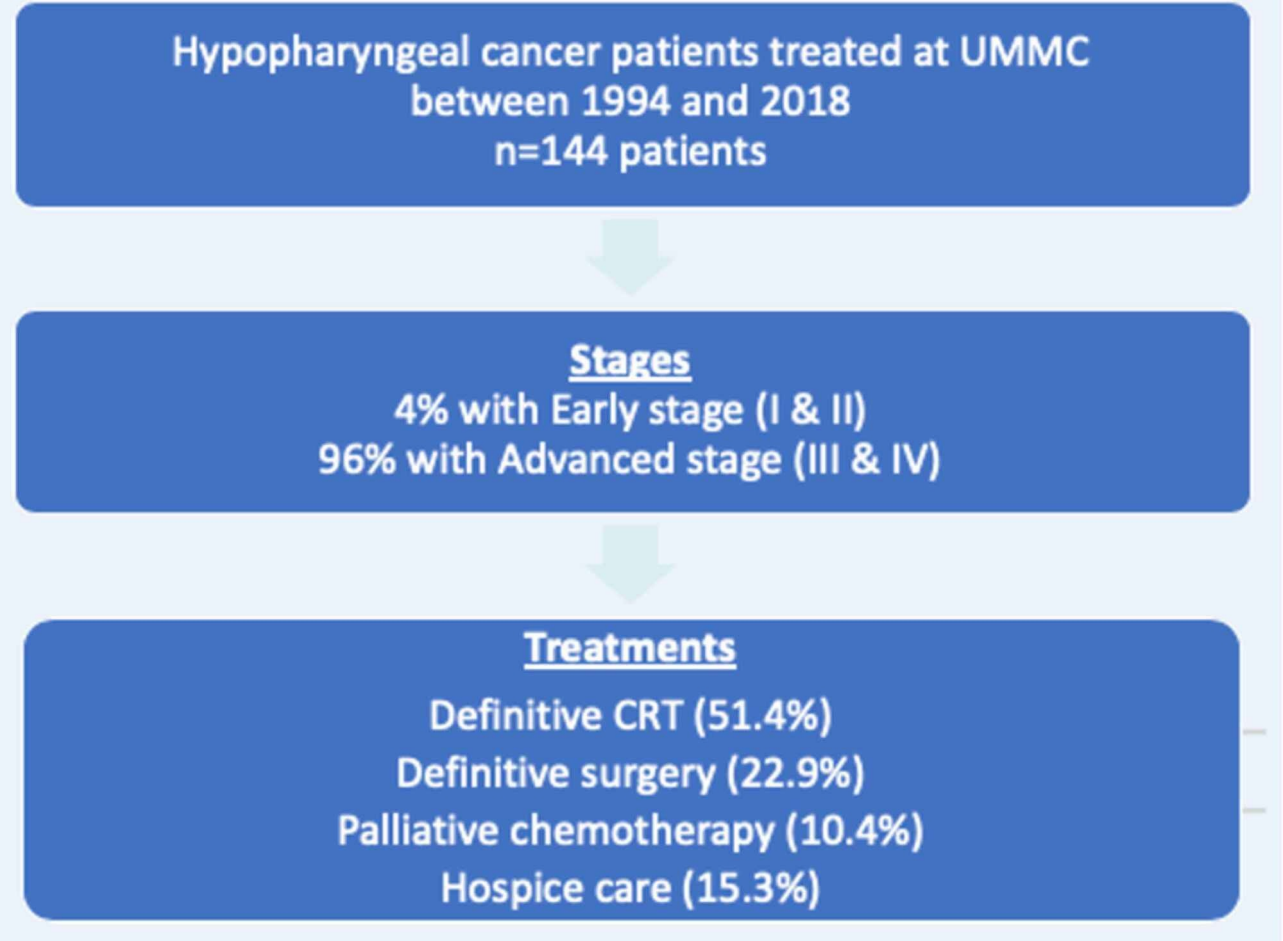
Hypopharyngeal cancer patients treated at UMMC between 1994 and 2018 UMMC: University of Mississippi Medical Center

One hundred forty-four hypopharyngeal carcinoma patients who were treated during the study period were identified (Figure 1). Our patient cohort consisted of 61.8 % AA and 35.4 % CA (P=0.538). Overall, 96% of them presented at an advanced stage (stages III & IV) of disease, and only 4% presented in the early stage (stages I & II). There was no significant difference between AA and CA presenting with advanced disease (96.6% vs. 94.1 %).

There was a significant difference in the insurance status of the patients. Among AA patients, 53.4% were on Medicaid while only 37.3% of CA patients had Medicaid. Regarding Medicare, 39.2% of CA patients had Medicare as compared with 15.9% of AA patients. Of the AA patients, 11.4% had private insurance as compared to 5.9% of CA patients. There was a significant difference between AA and CA patients who were classified as “self-pay” (19.3% vs. 17.6%, p=0.006). In our patient cohort, 15.3% of patients did not receive any therapy while 51.4%, 22.9%, and 10.4% of patients underwent definitive chemoradiotherapy, definitive surgery, or palliative chemotherapy, respectively. There were no significant differences within each treatment group between the two races.

**Figure 2:**
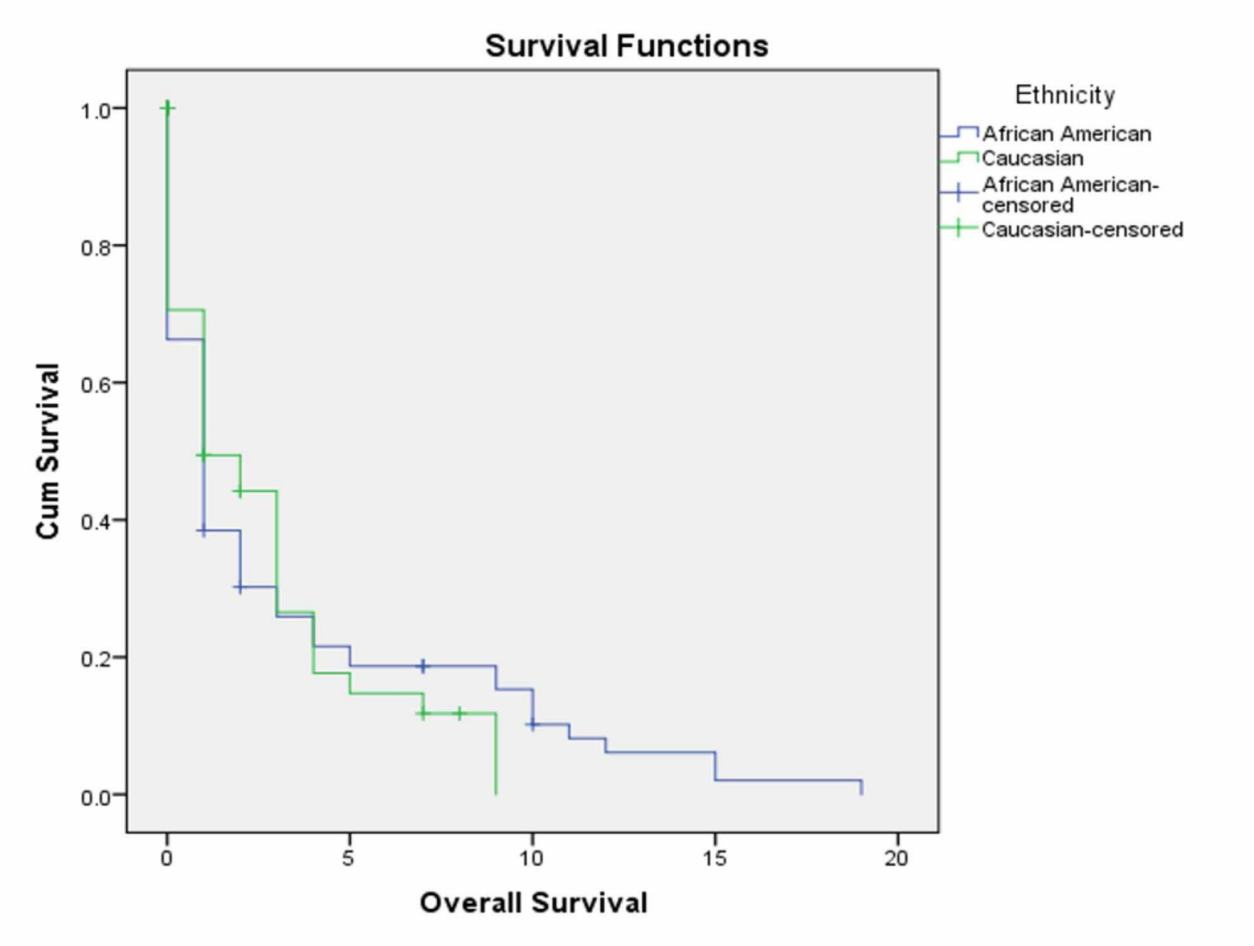
Kaplan-Meier overall survival for hypopharyngeal carcinoma patients by ethnicity/race

The median follow-up of the entire cohort was 13 months. Our analysis identified no significant difference in the median survival of AA and CA patients (16 months vs. 15 months; p=0.917). Besides, there appeared to be no significant differences in overall survival between AA and CA patients at three years or five years (Figure 2). These findings are summarized in Table 1.

**Table 1:** Demographic & clinical characteristics of the study population by race UNK: unknown; CRT: chemoradiotherapy

	Black (n=89; 61.8%)	White (n=51; 35.4%)	Others (n=4; 2.8%)	All (n=144)	p-value
Gender/Sex					
Male	85 (95.5%)	40 (78.4%)	4 (100%)	129 (89.5%)	0.005
Female	4 (4.5%)	11 (21.6%)	0 (0.0%)	15 (10.4%)	
Age					
≤50 years	21 (23.6%)	6 (11.8%)	0 (0.0%)	27 (18.8%)	0.14
> 50 years	68 (76.4%)	45 (88.2%)	4 (100%)	117 (81.3%)	
Insurance					
Medicaid	47 (53.4%)	19 (37.3%)	0 (0.0%)	66 (46.2%)	0.006
Medicare	14 (15.9%)	20 (39.2%)	1 (25.0%)	35 (24.5%)	
Private	10 (11.4%)	3 (5.9%)	2 (50.0%)	15 (10.5%)	
Self-pay	17 (19.3%)	9 (17.6%)	1 (25.0%)	27 (18.9%)	
Smoking Status					
Smoker	57 (64.0%)	38 (74.5%)	4 (100%)	99 (68.8%)	0.172
Non-Smoker	32 (36.0%)	13 (25.5%)	0 (0.0%)	45 (31.3%)	
Alcohol Status					
Drinker	47 (52.8%)	26 (51.0%)	3 (75.0%)	76 (52.8%)	0.651
Non-Drinker	42 (47.2%)	25 (49.0%)	1 (25.0%)	68 (47.2%)	
Subsite					
Pyriform Sinus	63 (70.8%)	36 (70.6%)	4 (100%)	103 (71.5%)	0.494
Post-Pharyngeal Wall	7 (7.9%)	7 (13.7%)	0 (0.0%)	14 (9.7%)	
Post-Cricoid	8 (9.0%)	1 (2.0%)	0 (0.0%)	9 (6.3%)	
Hypopharynx-UNK	11 (12.4%)	7 (13.7%)	0 (0.0%)	18 (12.5%)	
Overall Stage					
I	1 (1.1%)	1 (2.0%)	0 (0.0%)	2 (1.4%)	0.485
II	2 (2.2%)	2 (3.9%)	0 (0.0%)	4 (2.8%)	
III	14 (15.7%)	2 (3.9%)	1 (25.0%)	17 (11.8%)	
IV	72 (80.9%)	46 (90.2%)	3 (75.0%)	121 (84.0%)	
Treatment					
Definitive CRT	44 (49.4%)	29 (56.9%)	1 (25.0%)	74 (51.4%)	0.655
Definitive Surgery	19 (21.3%)	12 (23.5%)	2 (50.0%)	33 (22.9%)	
Palliative Chemo	11 (12.4%)	4 (7.8%)	0 (0.0%)	15 (10.4%)	
Hospice	15 (16.9%)	6 (11.8%)	1 (25.0%)	22 (15.3%)	

## Discussion

Incidence and prevalence

Hypopharyngeal cancer is a rare malignancy representing only 3% to 5% of head and neck cancers, with approximately 3,400 new cases diagnosed yearly in the United States. The estimated incidence and mortality of hypopharyngeal cancer in 2017 was 0.6 and 0.1 per 100,000 individuals, respectively [6]. The incidence of this disease has been gradually decreasing since the mid-twentieth century, likely secondary to the increased awareness and decreased usage of tobacco and alcohol [7]. The incidence has been decreasing by about 2.5% per year [6]. On the other hand, mortality rates have been gradually rising, perhaps secondary to this cancer being diagnosed in the more advanced stages due to its location and the longer symptomless progression of the disease [8]. Mortality has also been increasing by ~1.4 % per year [6].

The national incidence rate of hypopharyngeal cancer stands at 0.8 and 0.5 per 100,000 among AA and CA, respectively, and the mortality is 0.6 and 0.5 per 100,000 among AA and CA, respectively [6]. Although an overall decrease in incidence has been seen for this disease, a net 1.6% annual increase has been seen in AA females [6]. The reasons for the increased rate of hypopharyngeal cancer among AA females are most likely multifactorial. Research suggests this may be due to an increase in tobacco and alcohol use, poor nutrition, human papillomavirus (HPV) infections, workplace hazards, or genetic syndromes [7,9-10]. Although mixed reports suggest an increase in AA female incidence rates, recent mortality reports suggest a 1.2% decrease in AA and CA females diagnosed with hypopharyngeal cancer as opposed to the 1.9% increase in the disease for both AA and CA males [6]. Our analysis did not identify any racial differences in the incidence of hypopharyngeal cancers among AA and CA, which appears consistent with the majority of the literature on this subject.

Insurance status

The difference in insurance status and coverage varies among the two racial groups across the US. In a data analysis from the Medical Expenditure Panel Survey (MEPS), Berdahl et al. reported persistently lower rates of health insurance coverage among AA when compared to CA [11]. Another similar analysis conducted by Sohn with data from the Survey of Income Program Participation (SIPP) reported CA as representing the smallest proportion of uninsured patients as compared to the more substantial proportion of uninsured AA [12]. In cancer patients, AA patients were reportedly less likely to have private insurance and more likely to have Medicaid or Medicare [1]. It must be noted that beyond age 65, patients are eligible for Medicare, allowing for nearly universal health insurance coverage [12]. Our study found that a higher fraction of AA patients had Medicaid or were self-pay, which is consistent with the literature. Interestingly, however, a higher fraction of AA patients had private insurance as compared to CA among our patient population, which is contrary to the reported literature.

Disease stage at presentation

Regardless of racial differences, hypopharyngeal cancer patients are more commonly diagnosed at the distant stage or Stage III and Stage IV, the more advanced stages [6,13]. The late-stage presentation at diagnosis for patients may be due to the hypopharynx's anatomical location [4]. Since this structure is located below the oropharynx, it is visually inaccessible to a physician during a routine check-up [4]. A secondary cause in late-stage diagnosis among patients may also stem from the aggressive nature of cancer in which specific symptoms do not develop until advanced stages of the disease [13]. Our analysis was comparable to other studies as we also did not find a difference in the stage at the time of diagnosis between AA and CA. This is confirmed by 96% of the UMMC hypopharyngeal cancer patients diagnosed in the advanced stages and merely 4% of them diagnosed in the early stages.

Treatment

Even though advanced treatment options exist, the prognosis for advanced stage hypopharyngeal cancer is poor [14]. As multispecialty decision-making is required for optimal treatment selection, various factors are considered during a patient’s evaluation [14]. These include survival estimates, treatment benefits, adverse effects from treatment, patient expectations, and the patient’s quality of life [14-15]. There are minimal reports on racial disparities in the treatment of hypopharyngeal cancer among AA and CA patients. Zandberg et al. reported a multivariate regression analysis describing no difference in a hypopharyngeal cancer patient's survival after treatment, as survival was already lower as compared to other head and neck cancer subsites [10]. Kim et al. further described that most patients undergoing surgery also needed radiotherapy and chemoradiotherapy adjuvantly [14]. In these studies, health insurance, marital status, and even alcohol and tobacco consumption were viewed as factors impacting treatment decisions, leading potentially to differential treatment [14]. Our analysis was comparable to other studies, as we also did not find a difference in treatments administered between AA and CA patients.

Survival

Comparable data exist regarding racial disparities in survival among AA and CA patients. A Surveillance, Epidemiology, and Ends Results (SEER) database analysis identified 21% and 39% five-year survival rates for AA and CA, respectively [6]. Ragin et al. reported that AA and CA hypopharyngeal cancer patients had no significant relapse-free survival difference [1]. Moreover, Worsham et al. reviewed 358 hypopharyngeal patients as part of the Health Disparities Research Collaborative (HDRC) study and reported an absence of racial disparities in the stage of presentation and survival outcomes in AA and CA hypopharyngeal patients [16]. A multivariate Cox regression model conducted by Zandberg et al. also identified no difference in overall survival for AA and CA patients [10]. Similarly, in our retrospective analysis, we identified no significant difference in the overall survival between AA and CA patients at three years (27.2% vs. 36.3%; p=0.917) or five years (20.4% vs.16.7%; p=0.917).

Possible reasons for racial disparities

Although our study did not find any racial disparities in hypopharyngeal carcinoma patients, there have been multiple reports detailing the reasons for general cancer racial disparities in incidence and mortality. Guerrero-Preston et al. have proposed genetic factors predisposing AA patients to hypopharyngeal cancer outcomes [17]. Another study reported that general cancer care among low-income patients, despite access to care, included a lack of knowledge of resources, denial or fear, competing obligations, and embarrassment, potentially leading to diminished outcomes [9,18]. On the contrary, several other studies suggest that when hypopharyngeal cancer patients who mostly present at the advanced stage are given uniform patient care, racial disparities in disease prognosis and outcomes are not evident [1-4,8,10,16].

Limitations

The retrospective nature of and the modest number of patients in this study are two significant limitations. Confounding variables, such as medical comorbidities, which may have contributed to the observed survival differences, might have influenced our study results. As radiation and surgical techniques, along with systemic therapies, have vastly improved over the past 25 years, these changes may contribute to the observed patient results. Missing data in some of our patients included rescue treatments and death causality.

## Conclusions

A retrospective review of patients with hypopharyngeal cancer treated over a 25-year (1994-2018) period at a major academic medical center did not reveal significant racial disparity regarding the stage at diagnosis or prognosis. This study suggests that when patients have equal access to care, they are likely to have a similar prognosis despite racial differences. Further studies are needed to validate this hypothesis.
